# Risk factors of anaemia among postpartum women in Bolgatanga Municipality, Ghana

**DOI:** 10.1186/s40795-022-00550-7

**Published:** 2022-06-24

**Authors:** Anthony Wemakor, Alice Ziyaaba, Felix Yiripuo

**Affiliations:** Department of Nutritional Sciences, School of Allied Health Sciences, University for Development Studies, P. O. Box TL 1883, Tamale, Ghana

**Keywords:** Postpartum anaemia, Iron-folic acid, Tamale, Ghana

## Abstract

**Introduction:**

Anaemia is a major public health problem affecting women of reproductive age globally. This study was conducted to assess the prevalence and determinants of anaemia among postpartum women in Bolgatanga Municipality, Ghana.

**Methods:**

The study employed an analytical cross-sectional study design to recruit 405 women who delivered in the last 6 weeks from 9 health facilities in the Municipality. Data were collected on socio-demographic characteristics, obstetric characteristics, dietary diversity, knowledge on iron-folic acid (IFA), iron and anaemia, and haemoglobin level of the women. Postpartum anaemia (PPA) was defined as hemoglobin < 12 g/dl. Chi-square and logistic regression analysis were used to identify the determinants of PPA.

**Results:**

The mean age of the participants was 27.4 ± 5.3 years and 46.70% of them had PPA. The risk factors of PPA were not meeting dietary diversity [Adjusted Odds Ratio (AOR) = 2.96; 95% Confidence Interval (CI): 1.67–5.25], low knowledge on IFA, iron and anaemia (AOR = 3.03; 95% CI: 1.67–5.25), and first trimester pregnancy anaemia (AOR = 10.39; 95% CI: 1.32–6.95). Kusasi ethnicity was protective of PPA (AOR = 0.35; CI: 0.16–0.75). *Conclusion:* Anaemia is prevalent in postpartum women in Bolgatanga Municipality and its risk factors are dietary diversity, knowledge on IFA, iron and anaemia, pregnancy anaemia and ethnicity. Nutrition counselling and intervention in pregnancy and after delivery are warranted to reduce the burden of anaemia in this population.

## Introduction

Anaemia affects 56 million [1] or 38% of pregnant women [2] globally; pregnancy anaemia is an independent risk factor for postpartum anaemia (PPA) as iron stores tend to remain low for several months after childbirth [3, 4]. Globally, anaemia affects about 29% of non-pregnant women, and PPA affects 10—30% of mothers in developed countries [5] and 50—80% of mothers in less developed countries [6]. In Ghana, a study found that 16% of mothers has PPA in Volta Region [7].

In developing countries, at any life stage, anaemia is mainly caused by iron deficiency and deficiencies of vitamins A and B12, riboflavin, and folate; blood loss, parasitic infections and haemoglobinopathies [6, 8], and the same factors may be responsible for PPA. Postpartum women are susceptible to anaemia because of low maternal iron stores prior to and in pregnancy, blood loss during childbirth [9], iron use for breast milk production at the expense of maternal requirements [10], and poor iron supplementation in the postpartum period. Other risk factors of PPA include young maternal age [11], pregnancy anaemia [11, 12], postpartum haemorrhage [13], inadequate antenatal care visits [14], type of birth [12, 15], and poor adherence to iron and folic acid supplementation in pregnancy [16]. The consequences of PPA include tiredness, breathlessness, palpitations, reduced cognitive abilities, emotional instability, distress, increased risk of infections, and postpartum depression [5, 13, 17]. According to a meta-analysis, 20% of maternal mortality in sub-Saharan Africa and South Asia is attributable to iron deficiency anaemia [18] and PPA is a significant risk factor for maternal morbidity [19].

Postpartum care is one of the essential components of maternal and child health programmes but unfortunately it is poorly implemented in Ghana [20]. Prompt intervention in the postpartum period can prevent the deaths of both the mother and baby and reduce the risk of long-term pregnancy-related illnesses. Postpartum anaemia starts prior to or in pregnancy and efforts at preventing it must precede pregnancy. In line with the WHO’s recommendation to provide oral iron and/or folic acid supplementation to postpartum women for 6–12 weeks in settings where gestational anaemia is of public health concern [6], the Ghana Ministry of Health has been promoting iron supplementation to reduce the risk of anaemia in postpartum women.

Globally, most studies on anaemia in women of reproductive age concentrate on pregnant women and very few studies have been reported on the prevalence and risk factors of PPA. The situation in Ghana and specifically in the study area is not different and warrants an intervention. To plan for appropriate anaemia interventions, local evidence is crucial to inform healthcare professionals and other stakeholders to ensure optimal health for postpartum women and their infants. This study was therefore conducted to fill this gap in the literature and to provide baseline data for other researchers, policy makers, and implementers to influence planning and action on anaemia prevention. The main objective of the study was to determine the prevalence and determinants of anaemia among postpartum women in Bolgatanga Municipality, Ghana.

## Materials and methods

### Study design, site and population

The study was a cross-sectional facility-based study conducted in Bolgatanga Municipality. The Municipality is the regional capital and located in the centre of the Upper East Region, approximately between latitudes 10°30' and 10°50' North and longitudes 0°30' and 1°00' West. It covers a total land area of 729 square kilometers and is bordered to the north by the Bongo District, south and east by the Talensi and Nabdam Districts, and to the west by the Kassena-Nankana Municipality. The dominant tribe within the Municipality is Frafra and the dominant religions in the region are Christianity and Islam. The Bolgatanga Municipality has a total population of 131,550, accounting for 12.6% of the population of the Upper East Region, with females constituting 52.0% of the total population.

The study population included postpartum women aged 18–49 years and resident in the Municipality who delivered in the last 6 weeks and were available in the postnatal wards or were in attendance at the postnatal clinics of the participating health facilities at the time of the study. Women were interviewed if they were clinically stable with no active or symptomatic opportunistic infections and were willing to take part in the study.

### Sample size determination and sampling technique

The required sample size was determined using a single population proportion formula [21], assumed prevalence of anaemia in postpartum women in the area (50%), reliability coefficient associated with 95% confidence interval (1.96), and margin of error (5%) to obtain 385. Five percent of the estimated sample size (20) was added to obtain the final sample of 405.

The study participants were sampled from 9 health facilities in 9 sub-districts in the Municipality namely Bolgatanga Regional Hospital, Afrikids Medical Center, Plaza Health Center, Sherigu Health Center, Nyarega Health Center, Sumbrugu East Health Center, Ananega Health Center, Sumbrugu West CHPS, and Kalbeo CHPS.

The number of subjects sampled from each health facility was determined using probability proportional to size. On each visit to a health facility, thirty (30) postpartum women were randomly selected using balloting without replacement. This allowed consented participants to either pick “Yes” or “No” which was written on folded pieces of paper and placed in a container and thoroughly shaken to ensure randomization. Those who picked “Yes” were interviewed. The procedure was repeated until the total sample size was achieved.

### Data collection

The data collection took eight weeks from February—April, 2021 with the use of a semi-structured, interviewer-administered questionnaire in one-on-one interviews in health facilities. The drafted questionnaire was subjected to pre-testing and face validation to improve reliability and accuracy using 10 randomly picked women in War Memorial Hospital, Navrongo. Four research assistants who had knowledge on the research topic and had been in similar data collection exercises were trained to collect the data. Training on the questionnaire provided an opportunity for the research assistants to get a good understanding of the questions.

Data were collected on socio-demographic characteristics, obstetric characteristics, dietary diversity, haemoglobin in pregnancy and after delivery, and knowledge on iron-folic acid, iron, and anaemia of women. Socio-demographic data included: age in years, occupational status, marital status, education level, and parity status.

Data were collected on the knowledge of the women on iron-folic acid, iron, and anaemia. With iron-folic acid knowledge, the questions were on its benefits such as prevention of anaemia, protecting women from sickness, and making the foetus healthy. Others are the effects of iron and folic acid deficiencies such as a baby having congenital anomaly or being low birth weight, the dosage regimen of iron-folic acid for pregnant women, and side effects of iron-folic acid. 

#### Haemoglobin measurement

The two haemoglobin measurements in pregnancy of the women recorded in their ANC booklets were recorded and used to determine anaemia in pregnancy. In the postpartum period, the research project measured the haemoglobin concentration of the women using two experienced laboratory technicians. The haemoglobin estimation was determined by taking finger-pricked blood test samples of participants using URIT-12 Haemoglobin photometer (URIT Medical Electronics Co., LTD, China). The haemoglobin values displayed on the Haemoglobin photometer were recorded.

### Definition of study variables

#### Dependent variable

Anaemia in postpartum women: There is no consensus on the definition of anaemia in postpartum women. However, we defined anaemia in postpartum women with haemoglobin less than 12 g/dl [5].

#### Independent variables

##### Anaemia in pregnancy

Anaemia in the first and third trimesters of pregnancy was diagnosed using a cut-off of haemoglobin < 11 g/dl [22].

##### Household Wealth Index

A household wealth index of the respondents was obtained based on the availability of electricity, water, and toilets in the households, possession of household items (e.g., bicycles, television, and radio), and livestock in the household based on an earlier concept [23]. Using principal component analysis, a wealth score was derived for the respondents, sorted in ascending order and divided into 3 categories, poorest, middle, and richest.

##### Minimum Dietary Diversity—Women (MDD-W)

Dietary diversity score was calculated from 10 designated food groups [24]. For each food group, the women ate from, they got a score of “1” (irrespective of the number of foods eaten), otherwise a score of “0”. The scores were added up to give the dietary diversity score (range 0 – 10) for each woman. Using the dietary diversity score, an indicator variable, MDD-W, was obtained. Women who had a dietary diversity score of 5 or more were classified as having received MDD-W, otherwise they did not receive it. The proportion of women receiving MDD-W at the population level is an indicator of higher micronutrient adequacy [24].

##### IFA knowledge index

An IFA knowledge index was constructed using responses from 18 questions. Each correct answer attracted a score of “1” otherwise a score of “0”. The scores were totaled and divided into two halves using the mean score; women scoring lower than the mean score (7.2 out of 18.0) were classified into the low category and those scoring the mean score or higher into the high category.

##### Iron knowledge index

Iron knowledge index was based on knowledge of the mothers of 6 food sources of iron and divided into high and low categories using the mean score of 2.5 out of 6.

##### Anaemia knowledge index

Similarly, the anaemia knowledge index was constructed from 6 questions on sign/symptoms of anaemia and divided into two halves, high and low, using the mean score (2.5).

##### Composite index of IFA, iron, and anaemia

Using the scores of IFA knowledge, iron knowledge, and anaemia knowledge, a composite maternal nutrition knowledge index was constructed with scores ranging theoretically from 0–30. The mean score of 12.3 was used to divide the index into high (≥ 12.3) and low (< 12.3) categories.

### Data analysis

The questionnaire was double-checked for completeness and accuracy before entry into the software for data analysis. The data were analysed using Stata (Stata Corps, College Road, Texas). Descriptive and inferential statistics were used to present the results. Bivariate tests with Chi-square test of independence were carried out and statistically significant factors were entered into a logistic regression model to yield adjusted odds ratios with 95% confidence intervals for the identification of risk factors for postpartum anaemia. The fit of the model was evaluated using Hosmer–Lemeshow goodness of fit test. Multicollinearity among the independent variables was checked using the “collin” command in Stata. All statistical tests were performed using two-sided tests at the 0.05 level of significance.

### Ethics approval and consent to participate

The methodology of this research conformed to the ethical principles of the Helsinki Declaration, and ethics approval was obtained from the Committee on Human Research, Publication and Ethics of Kwame Nkrumah University of Science and Technology and Komfo Anokye Teaching Hospital, Kumasi, Ghana (CHRPE/AP/063/21). Moreover, permission was sought from Bolgatanga Municipal Health Directorate, Ghana. Written informed consent was obtained from participants before they participated in this study. No person identifiable information was collected and confidentiality of the information of study participants was assured.

## Results

### Socio-demographic and obstetric characteristics of postpartum women

Four hundred and five (405) postpartum women were surveyed. The mean age of the respondents was 28.7 ± 4.7 years and most were in the age group 31 + years (37.5%). About a third of the respondents (31.4%) had tertiary level education but the majority were married (88.9%) and most (64.2%) were Christians, Table 1. The results revealed that the largest proportion of the participants (29.4%) were petty traders/vendors, 14.8% were housewives and unemployed, and the majority were of the Frafra ethnic group (60.7%). The women were distributed evenly by wealth index.

**Table 1:** Socio-demographic, obstetric and dietary characteristics of postpartum women (*n* = 405)

Variable category	Frequency	Percent
Maternal age (years)		
18–25	108	26.7
26–30	145	35.8
31 +	152	37.5
Education		
None	83	20.5
Primary	77	19.0
Secondary	118	29.1
Tertiary	127	31.4
Marital status		
Single	27	6.7
Married	360	88.9
Separated	18	4.4
Religion		
Christian	260	64.2
Islamic	121	29.9
Others	24	5.9
Occupation		
Trader/vendor	119	29.4
Agricultural or service worker	111	27.4
Office, educational or health worker	115	28.4
Housewife	60	14.8
Ethnicity		
Frafra	246	60.7
Kusaasi	66	16.3
Builsa or Talensi	51	12.6
Others	42	10.4
Household wealth tertile		
Poorest	137	33.8
Medium	140	34.6
Richest	128	31.6

### Obstetric and dietary characteristics of postpartum women

About half of the women have had 3 or more pregnancies (46.2%) or births (44.7%), Table 2. Majority of the women delivered naturally (85.7%), and had a birth interval of at least 24 months (70.9%). Majority of women reported taking IFA regularly in pregnancy (94.3%) and receiving counselling on IFA (94.8%). The incidence of low birth weight (birth weight < 2.5 kg) was low 5.7% (23/405). All postpartum women (100%) indicated that they had consumed grains, white roots, and tubers, whereas nuts and seeds was the least 25.2% (102/405) consumed food group by the women. About a third of the postpartum women, 35.6% (144/405) did not receive minimum dietary diversity.

**Table 2:** Obstetric and dietary characteristics of postpartum women (*n* = 405)

Variable	Frequency	Percent (%)
Gravidity		
1	113	27.9
2	105	25.9
3 +	187	46.2
Parity		
1	109	26.9
2	115	28.4
3 +	181	44.7
Inter-pregnancy interval		
< 24 months	118	29.1
> = 24 months	287	70.9
Mode of delivery		
Caesarean section	58	14.3
Spontaneous vaginal delivery	347	85.7
Regularly take IFA supplement in pregnancy		
No	23	5.7
Yes	382	94.3
Received counselling on IFA		
No	21	5.2
Yes	384	94.8
Low birth weight		
No	382	94.3
Yes	23	5.7
Minimum dietary diversity – women		
No	144	35.6
Yes	261	64.4

### Knowledge of postpartum women on IFA

The knowledge of the women was examined on benefits, daily dose, duration of use, side effects, and management of side effects of IFA. A little less than half of the women (45.2 – 48.1%) were able to identify two benefits of IFA to mothers and babies with 48.1% saying it increases the amount of blood in women, Table 3. Apart from darkening of faeces (40.5%), about a third of the mothers (32.8—33.1%) knew at least one side effect of IFA and a similar proportion mentioned at least one way of managing the side effects (37.8 – 38.0%). A little more than half knew the right daily dosing of IFA given to them by the health facilities and the duration of IFA usage advised. About 40.0% of the women could mention one of the four deficiency signs/symptoms of IFA. Combining these variables into a composite index for IFA, 60.0% of the women had low knowledge on IFA.

**Table 3:** Knowledge of postpartum women on IFA, iron and anaemia (*n* = 405)

Variable category	Frequency	Percent
Knowledge of benefits of IFA		
Protects mother from sicknesses	184	45.4
Gives mother strength during delivery	183	45.2
Increases amount of blood	195	48.1
Makes foetus grow healthy and strong	187	46.2
Frequency of intake of IFA	**205**	**50.6**
Duration of intake of IFA	**208**	**51.4**
Knowledge of side effects of IFA		
Epigastric pain	134	33.1
Abdominal pain	134	33.1
Nausea	135	33.3
Vomiting	135	33.3
Diarrhoea	133	32.8
Constipation	133	32.8
Faeces may turn black	164	40.5
Knowledge on managing the side effects of IFA		
Take IFAS with meals	154	38.0
Take IFAS while going to bed	153	37.8
Knowledge of consequences of IFA deficiency		
Excessive bleeding during pregnancy/delivery	164	40.5
Low birth weight baby	166	41.0
Preterm baby	165	40.7
IFA knowledge index		
Low	243	60.0
High	162	40.0
Knowledge of rich sources of iron		
Liver	168	41.5
Red meat e.g. beef	170	42.0
White meat e.g. chicken, fish	164	40.5
Dark-green leafy vegetables	169	41.7
Legumes e.g. beans, peas	176	43.5
Eggs	175	43.2
Iron knowledge index		
Low	232	57.3
High	173	42.7
Knowledge of signs/symptoms of anaemia		
Looks pale	179	44.2
Palpitations	165	40.7
Headaches	167	41.2
Dizziness	172	42.5
Tiredness and easily fatigued	164	40.5
Swells legs	165	40.7
Anaemia knowledge index		
Low	234	57.8
High	171	42.2
IFA, iron and anaemia knowledge index		
Low	239	59.0
High	166	41.0

### Knowledge of postpartum women on iron

Knowledge of women on iron focused on its rich sources. About 40.0% of the women were able to identify one rich source such as liver, meat, dark green leafy vegetables, legumes, and eggs. Combining the data on these variables into a composite index for iron, 57.3% of the mothers had low knowledge on iron.

### Knowledge of postpartum women on anaemia

Anaemia knowledge was examined using 6 signs/symptoms of anaemia. About 40% of the respondents knew at least one of the signs/symptoms of anaemia such as paleness, heart palpitations, headaches, dizziness, and tiredness/easy fatigability. Deriving a composite index for anaemia from these, 57.8% of the mothers had low knowledge on anaemia.

### Composite index of IFA, iron, and anaemia

A composite index was constructed using IFA, iron, and anaemia knowledge indices. Based on the composite index, 59.0% of the women had low knowledge.

### Prevalence of anaemia among postpartum women

Two haemoglobin measurements in pregnancy done by the health facilities and recorded on the ANC booklets were recorded and used to estimate anaemia prevalence in pregnancy, Table 4. The mean of the first haemoglobin measurement was 11.36 g/dl with a range of 7.9–14.6 g/dl and the mean of the second measurement was 11.38 g/dl (range: 6.0–15.7 g/dl). Based on the first and second haemoglobin measurements, 29.9% and 40.0% of the women were anaemic. Majority of the women were in their first (85.2%) and third (89.1%) trimesters for the first and second haemoglobin measurements. Hemoglobin measurements during the survey in the postpartum period found 46.7% of the women to have anaemia.

**Table 4:** Haemoglobin measurements and anaemia prevalence during pregnancy and postpartum period (*n* = 405)

Haemoglobin (g/dl)			Anaemia	Frequency	Percent
**First trimester pregnancy**	Mean	11.36	No	284	70.1
	Range	7.9–14.6	Yes	121	29.9
**Third trimester pregnancy**	Mean	11.38	No	243	60.0
	Range	6.0–15.7	Yes	162	40.0
**Postpartum period**	Mean	11.84	No	216	53.3
	Range	8.2–16.2	Yes	189	46.7

Comparing anaemia prevalence in pregnancy and postpartum period, 26.6% (70) of those not having anaemia in the first trimester had developed anaemia in the third trimester, and 50.6% (44) of those who were not anaemic in the third trimester (*n* = 214), developed anaemia in the postpartum period, Table 5. However, of those who were anaemic during the first trimester of pregnancy, 24.0% (29) had recovered at the time of the third trimester, and 12.0% (11) of those anaemic in the third trimester (*n* = 92), had recovered in the postpartum period.

**Table 5:** Comparison of anaemia prevalence in pregnancy and postpartum period (*n* = 405)

First trimester pregnancy anaemia	Frequency	Third trimester pregnancy anaemia	Frequency (%)	Postpartum anaemia	Frequency (%)
**No**	284	No	214 (75.4)	No	170 (79.4)
				Yes	44 (50.6)
		Yes	70 (26.6)	No	11 (37.9)
				Yes	18 (62.1)
**Yes**	121	No	29 (24.0)	No	11 (37.9)
				Yes	18 (62.1)
		Yes	92 (76.0)	No	11 (12.0)
				Yes	81 (88.0)

### Bivariate analysis of risk factors of anaemia among postpartum women

At the bivariate level, there was a significant association between postpartum anaemia and education (*p* < 0.001), religion (*p* = 0.043), occupation (*p* < 0.001), ethnicity (*p* = 0.040), household wealth index (*p* < 0.001), inter-pregnancy interval (*p* = 0.029), minimum dietary diversity-women (*p* < 0.001), IFA knowledge index (*p* < 0.001), anaemia knowledge index (*p* < 0.001), and composite IFA, iron and anaemia knowledge index (*p* < 0.001), Table 6. Other significant factors were first trimester pregnancy anaemia (*p* < 0.001) and third trimester pregnancy anaemia (*p* < 0.001).

**Table 6:** Risk factors for postpartum anaemia (bivariate analysis) (*n* = 405)

Variable	Total	Postpartum Anaemia		Test Statistics
		**No, Freq (%)**	**Yes, Freq (%)**	
**Maternal age (years)**				X2 = 2.1; *p* = 0.350
** 18–25**	108	52 (48.1)	56 (51.9)	
** 26–30**	145	77 (53.1)	68 (46.9)	
** 31 + **	152	87 (57.2)	65 (42.8)	
**Education**				X2 = 36.5; *p* < 0.001
** None**	83	33 (39.8)	50 (60.2)	
** Primary**	77	39 (50.6)	38 (49.4)	
** Secondary**	118	49 (41.5)	69 (58.5)	
** Tertiary**	127	95 (74.8)	32 (25.2)	
**Marital status**				X2 = 5.2; *p* = 0.076
** Single**	27	11 (40.7)	16 (59.3)	
** Married**	360	199 (55.3)	161 (44.7)	
** Separated**	18	6 (33.3)	12 (66.7)	
**Religion**				X2 = 6.3; *p* = 0.043
** Christian**	260	150 (57.7)	110 (42.3)	
** Islamic**	121	57 (47.1)	64 (52.9)	
** Others **	24	9 (37.5)	15 (62.5)	
**Occupation**				X2 = 33.7; *p* < 0.001
** Trader**	119	57 (47.9)	62 (52.1)	
** Agricultural and service worker**	111	53 (47.7)	58 (52.3)	
** Office, educational and health worker**	115	86 (74.8)	29 (25.2)	
** Housewife**	60	20 (33.3)	40 (66.7)	
**Ethnicity**				X2 = 8.3; *p* = 0.040
** Frafra**	246	131 (53.3)	115 (46.7)	
** Kusaasi**	66	44 (66.7)	22 (33.3)	
** Bulisa or Talansi**	51	21 (41.2)	30 (58.8)	
** Others**	42	20 (47.6)	22 (52.4)	
**Gravidity**				X2 = 1.1; *p* = 0.289
** 1–3**	320	175 (54.7)	145 (45.3)	
** 4 + **	85	41 (48.2)	44 (51.8)	
**Parity**				X2 = 0.3; *p* = 0.590
** 1–4**	384	206 (53.6)	178 (46.4)	
** 5 + **	21	10 (47.6)	11 (52.4)	
**Household wealth tertile**				X2 = 33.6; *p* < 0.001
** Poorest**	137	48 (35.0)	89 (65.0)	
** Medium**	140	78 (55.7)	62 (44.3)	
** Richest**	128	90 (70.3)	38 (29.7)	
**Inter-pregnancy interval**				X2 = 4.7; *p* = 0.029
**< 24 months**	118	53 (44.9)	65 (55.1)	
**> = 24 months**	287	163 (56.8)	124 (43.2)	
**Mode of delivery**				X2 = 0.1; *p* = 0.791
** Caesarean section**	58	30 (51.7)	28 (48.3)	
** Spontaneous vaginal delivery**	347	186 (53.6)	161 (46.4)	
**Minimum dietary diversity – women**				X2 = 52.4; *p* < 0.001
** No**	144	42 (29.2)	102 (70.8)	
** Yes**	261	174 (66.7)	87 (33.3)	
**IFA knowledge index**				X2 = 42.9; *p* < 0.001
** Low**	237	94 (39.7)	143 (60.3)	
** High**	168	122 (72.6)	46 (27.4)	
**Anaemia knowledge index**				X2 = 41.1; *p* < 0.001
** Low**	234	93 (39.7)	141 (60.3)	
** High**	171	123 (71.9)	48 (28.1)	
**Composite index of IFA, iron and anaemia**				X2 = 43.2; *p* < 0.001
** Low**	239	95 (39.7)	144 (60.3)	
** High**	166	121 (72.9)	45 (27.1)	
**First trimester pregnancy anaemia**				X2 = 85.7; *p* < 0.001
** No**	284	194 (68.3)	90 (31.7)	
** Yes**	121	22 (18.2)	99 (81.8)	
**Third trimester pregnancy anaemia**				X2 = 109.2; *p* < 0.001
** No**	243	181 (74.5)	62 (25.5)	
** Yes**	162	35 (21.6)	127 (78.4)	
**Regularly takes IFA in pregnancy**				X2 = 0.0; *p* = 0.909
** No**	23	12 (52.2)	11 (47.8)	
** Yes**	382	204 (53.4)	178 (46.6)	

### Multivariate analysis of risk factors of anaemia among postpartum women

The multivariate analysis showed that the independent determinants of anaemia in postpartum women in Bolgatanga Municipality were minimum dietary diversity-women, composite index of IFA, iron, and anaemia knowledge, first pregnancy anaemia and ethnicity, Table 7. Women who did not take minimum dietary diversity were about 3 times more likely to be anaemic compared to those who had taken it [Adjusted Odds Ratio (AOR) = 2.96; 95% Confidence Interval (CI): 1.67–5.25; *p* < 0.001]. Similarly, women belonging to the low composite knowledge index for IFA, iron, and anaemia were 3 times more likely to be anaemic compared to those belonging to the high category of the composite index (AOR = 3.03; 95% CI: 1.67–5.25; *p* < 0.001). The strongest risk factor for anaemia in the postpartum period is pregnancy anaemia. Women who were anaemic in the first trimester pregnancy were 10 times more likely to be anaemic in the postpartum period (AOR = 10.39; 95% CI: 1.32–6.95; *p* = 0.009). Moreover, ethnicity of the women emerged as an independent determinant of anaemia in the postpartum period; Kusaasi women were 65% protected from anaemia compared to Frafras (AOR = 0.35; CI: 0.16–0.75; *p* = 0.007). The Hosmer–Lemeshow goodness of fit test showed that the logistic regression model fitted the data well (*p* = 0.1113).

**Table 7:** Multivariate analysis of factors associated with anaemia among postpartum women (*n* = 405)

Characteristics	Adjusted Odds Ratio	95% Confidence Interval	*P*-value
Postpartum anaemia			
Education			
None	1.45	0.42–4.98	0.560
Primary	1.04	0.30–3.65	0.946
Secondary	1.79	0.61–5.20	0.287
Tertiary	1.00		
Religion			
Christian	1.00		
Islamic	1.74	0.94–3.24	0.080
Others	2.62	0.87–7.86	0.087
Occupation			
Trader	0.47	0.16–1.44	0.189
Agricultural or service worker	0.70	0.22–2.23	0.546
Office, educational or health worker	1.00		
Housewife	0.70	0.21–2.28	0.551
Ethnicity			
Frafra	1.00		
Kusaasi	0.35	0.16–0.75	0.007
Builsa or Talensi	1.59	0.72–3.49	0.250
Others	1.48	0.64–3.40	0.361
Household wealth tertile			
Poorest	0.92	0.42–2.01	0.828
Medium	0.81	0.39–1.66	0.559
Richest	1.00		
Inter-pregnancy interval			
< 24 months	1.49	0.84–2.64	0.173
> = 24 months	1.00		
Minimum dietary diversity – women			
No	2.96	1.67–5.25	< 0.001
Yes	1.00		
Composite knowledge index of IFA, iron and anaemia			
Low	3.03	1.32–6.95	0.009
High	1.00		
First trimester pregnancy anaemia			
No	1.00		
Yes	10.39	5.65–19.11	< 0.001

## Discussion

This hospital-based, cross-sectional study determined the magnitude of anaemia and its determinants in postpartum women in Bolgatanga Municipality, Ghana. The study found that anaemia in postpartum women is of public health significance and its strongest risk factor is first trimester pregnancy anaemia.

The magnitude of PPA in our study population based on haemoglobin < 12 g/dl (46.7%) is higher than the prevalence reported for a group of postpartum women in Hohoe, Ghana, (16%) [7] using a cut-off of haemoglobin < 10 g/dl. Our estimate is higher than the magnitude of anaemia reported for postpartum women in Ethiopia, 22.1% among women in a nationally representative study using haemoglobin < 12 g/dl [25] and 29% in another study using haemoglobin < 10 g/dl [18]; and in Tanzania, 21.6%, using haemoglobin < 11 g/dl [26]. The rate in the study population is however comparable to the rate for postpartum women in Kenya (43.8%) with haemoglobin < 12 g/dL [27] but less than what pertains in some Asia countries e.g., 60.3% in Myanmar [28], and 66.0% [29], and 76.2% [30] in India. These rates are difficult to compare as the studies employed different definitions of anaemia owing to a lack of international agreement on anaemia definition in postpartum women. In addition to differences in anaemia cut-offs, postpartum periods, diagnostic procedures, populations studied, and socio-demographic and biomedical factors may explain the differences in the PPA rates measured. According to WHO, PPA is a severe public health problem in the population studied as more than 40% of the population is affected [31] warranting immediate intervention. However, it is imperative to institute measures that timely identify women at risk of anaemia or have anaemia in pregnancy and after childbirth for timely and appropriate intervention. An innovative screening protocol implemented in Israel that led to a 22% increase in the diagnosis of moderate-severe anaemia [32] could be adapted and used in the study area to improve anaemia diagnosis.

Risk factors for PPA in the population studied include minimum dietary diversity-women, IFA, iron and anaemia knowledge index, pregnancy anaemia, and ethnicity. We found that anaemia was more prevalent in women of low dietary diversity; in a similar study to ours, anaemia among postpartum women was found to be associated with low dietary diversity score (DDS) in Tanzania [33]. Consumption of minimum dietary diversity is more likely to ensure the availability of non-haem iron, haem iron, and ascorbic acid, providing the necessary ingredients for haematopoiesis. This finding suggests that, food diversification may help to reduce the risk of anaemia and presupposes that the promotion of dietary diversity by eating foods from at least five groups should be given priority to reduce anaemia and other forms of undernutrition.

Mothers with inadequate knowledge on IFA, iron, and anaemia had a higher likelihood of getting anaemia. It is possible that women with adequate knowledge on benefits and deficiency signs/symptoms of IFA and who know iron-rich food sources are more likely to use IFA or iron supplements, consume iron-rich foods, and detect and promptly treat anaemia. Results from a meta-analysis showed that pregnant women who had knowledge on IFA supplementation or anaemia were more likely to comply with IFA supplementation in pregnancy compared to those who had limited knowledge on IFA or anaemia [34]. Enhanced knowledge of women on ways of identifying risk factors related to anaemia will reduce anaemia during pregnancy and in the postpartum period. Health education and promotion messages on iron, folic acid, and anaemia among pregnant and postpartum women should be delivered in a culturally acceptable manner using nutrition behavior change communication strategies. It is when women are equipped with the needed knowledge that they can prevent the occurrence of anaemia. Educating and providing women with livelihoods will empower them to be able to purchase and eat adequate nutritious foods that would enhance their nutritional and haemoglobin status.

Anaemia magnitude increased from first trimester pregnancy to the postpartum period and the strongest risk factor for PPA is first trimester pregnancy anaemia. Similarly, a study found third trimester pregnancy anaemia to be a risk factor of postpartum anaemia [11]. Anaemia is difficult to correct, so preexisting anaemia before pregnancy, or anaemia in pregnancy may be carried over into the postpartum period.

We also found that women belonging to one ethnic group are protected from anaemia in the postpartum period compared to those of another ethnic group. The likely reasons for this observation are unknown, so further studies are warranted to explain it.

### Strengths and limitations

Most studies on haemoglobin status of women concentrate on pregnant women, but a particular strength of this study is the assessment of haemoglobin status in both pregnancy and postpartum period in a cohort of mothers. Even though the study reports on the prevalence of anaemia in pregnancy and postpartum period, the haemoglobin measurements in pregnancy were recorded from the ANC cards and not estimated by the researchers, unlike the haemoglobin measurement for the postpartum period. Like any cross-sectional, questionnaire-based, observational study, the study investigated association and not causation and cannot rule out recall bias as it relied on the responses of the mothers. Finally, postpartum haemorrhage, which is an important determinant of postpartum anaemia, was not measured. Despite these limitations, we believe that this study provides important baseline data on postpartum anaemia in Ghana.

## Conclusions

Anaemia is prevalent in postpartum women and its risk factors are minimum dietary diversity, knowledge on IFA, iron and anemia, pregnancy anaemia and ethnicity. Nutrition counselling and intervention in pregnancy and after delivery are warranted to reduce the burden of anaemia in this population. Currently, most ante- and postnatal clinics in small health facilities in Ghana are managed by health care providers who may not be adequately trained to offer individualized medical nutrition therapy to pregnant and lactating mothers. Nutritionists and dietitians should be assigned at all levels of the health system, especially for maternal and child health services, to promote a multidisciplinary approach in the prevention and management of anaemia. Frontline health care workers providing antenatal care services should receive regular in-service training to improve nutrition screening, assessment, and interventions to prevent and manage anaemia among pregnant and postpartum women.

## Data Availability

The datasets used and/or analysed during the current study are available from the corresponding author on reasonable request.

## Abbreviations

ANC: Antenatal Clinic
AOR: Adjusted Odds Ratio
CHPS: Community-based Health Planning and Services
CI: Confidence Interval
IFA: Iron and Folic Acid
IPT: Intermittent Preventive Treatment
MDD-W: Minimum Dietary Diversity-Women
PPA: Postpartum Anaemia
WHO: World Health Organisation

## References

[1] Balarajan Y, Ramakrishnan U, Ozaltin E, Shankar AH, Subramanian SV (2011). Anaemia in low-income and middle-income countries. Lancet (London, England).

[2] Stevens GA, Finucane MM, De-Regil LM, Paciorek CJ, Flaxman SR, Branca F, Peña-Rosas JP, Bhutta ZA, Ezzati M (2013). Global, regional, and national trends in haemoglobin concentration and prevalence of total and severe anaemia in children and pregnant and non-pregnant women for 1995–2011: a systematic analysis of population-representative data. Lancet Glob Health.

[3] Bodnar LM, Cogswell ME, Scanlon KS (2002). Low income postpartum women are at risk of iron deficiency. J Nutr.

[4] Reveiz L, Gyte GM, Cuervo LG, Casasbuenas A. Treatments for iron-deficiency anaemia in pregnancy. Cochrane Database Syst Rev. 2011(10):Cd003094. 10.1002/14651858.CD003094.pub3 PMC12989263 21975735

[5] Milman N (2011). Postpartum anemia I: definition, prevalence, causes, and consequences. Ann Hematol.

[6] World Health Organization (2016). Guideline: Iron supplementation in postpartum women.

[7] Kofie P, Tarkang EE, Manu E, Amu H, Ayanore MA, Aku FY, Komesuor J, Adjuik M, Binka F, Kweku M (2019). Prevalence and associated risk factors of anaemia among women attending antenatal and post-natal clinics at a public health facility in Ghana. BMC nutrition.

[8] Camaschella C (2015). Iron-deficiency anemia. N Engl J Med.

[9] Whitney E, Rolfes SR (2018). Understanding nutrition: Cengage Learning.

[10] Domellöf M, Lönnerdal B, Dewey KG, Cohen RJ, Hernell O (2004). Iron, zinc, and copper concentrations in breast milk are independent of maternal mineral status. Am J Clin Nutr.

[11] Rakesh P, Gopichandran V, Jamkhandi D, Manjunath K, George K, Prasad J (2014). Determinants of postpartum anemia among women from a rural population in southern India. Int J Women's Health.

[12] Pratiwi IR, Santoso S, Wahyuningsih HP (2018). Prevalence and risk factors for postpartum anemia. Jurnal Kesehatan Ibu dan Anak.

[13] Bergmann RL, Richter R, Bergmann KE, Dudenhausen JW (2010). Prevalence and risk factors for early postpartum anemia. Eur J Obstet Gynecol Reprod Biol.

[14] Alemayehu M. Factors associated with anemia among lactating mothers in subsistence farming households from selected districts of Jimma zone, south western Ethiopia: a community based cross-sectional study. J Nutr Food Sci. 2017;7(3):595.

[15] Darmawati D, Syahbandi S, Fitri A, Audina M. Prevalence and Risk Factors of Iron Deficiency Anemia among Postpartum Women. J Nurs Care. 2020;3(3):206-11.

[16] Nawagi F. Incidence and Factors Associated with Postpartum Anemia at Mbarara Regional Referral Hospital. Mortality. 2016;23:37–47.

[17] Beard JL, Hendricks MK, Perez EM, Murray-Kolb LE, Berg A, Vernon-Feagans L, Irlam J, Isaacs W, Sive A, Tomlinson M (2005). Maternal iron deficiency anemia affects postpartum emotions and cognition. J Nutr.

[18] Ross J, Thomas E. Iron deficiency anemia and maternal mortality. Working Notes Series. 1996;3.

[19] World Health Organization. WHO recommendations on postnatal care of the mother and newborn. Geneva: World Health Organization; 2014. 24624481

[20] Browne JL, Kayode GA, Arhinful D, Fidder SA, Grobbee DE, Klipstein-Grobusch K (2016). Health insurance determines antenatal, delivery and postnatal care utilisation: evidence from the Ghana Demographic and Health Surveillance data. BMJ Open.

[21] Snedecor G, William G (1989). Statistical methods/george w. Snedecor and william g Cochran.

[22] World Health Organization. Haemoglobin concentrations for the diagnosis of anaemia and assessment of severity. Geneva: World Health Organization; 2011.

[23] Garenne M, Hohmann-Garenne S (2003). A wealth index to screen high-risk families: application to Morocco. J Health Popul Nutr.

[24] Fao F (2016). Minimum dietary diversity for women: a guide for measurement.

[25] Lakew Y, Biadgilign S, Haile D (2015). Anaemia prevalence and associated factors among lactating mothers in Ethiopia: evidence from the 2005 and 2011 demographic and health surveys. BMJ Open.

[26] Tairo SR, Munyogwa MJ (2022). Maternal anaemia during postpartum: preliminary findings from a cross-sectional study in Dodoma City, Tanzania. Nurs Open.

[27] Ettyang G, van Marken LW, Oloo A, Saris W (2003). Serum retinol, iron status and body composition of lactating women in Nandi, Kenya. Ann Nutr Metabol.

[28] Zhao A, Zhang Y, Li B, Wang P, Li J, Xue Y, Gao H (2014). Prevalence of anemia and its risk factors among lactating mothers in Myanmar. Am J Trop Med Hyg.

[29] Singh A, Kandpal S, Chandra R, Srivastava V, Negi K (2009). Anemia amongst pregnant and lactating women in district Dehradun. Indian J Prev Soc Med.

[30] Selvaraj R, Ramakrishnan J, Sahu SK, Kar SS, Laksham KB, Premarajan K, Roy G (2019). High prevalence of anemia among postnatal mothers in Urban Puducherry: a community-based study. J Fam Med Primary Care.

[31] WHO W: The global prevalence of anaemia in 2011. Geneva: World Health Organization 2015.

[32] Yefet E, Suleiman A, Garmi G, Hatokay A, Nachum Z (2019). Evaluation of postpartum anaemia screening to improve anaemia diagnosis and patient care: a prospective non-randomized before-and-after anaemia screening protocol implementation study. Sci Rep.

[33] Tairo SR, Munyogwa MJ (2022). Maternal anaemia during postpartum: preliminary findings from a cross-sectional study in Dodoma City, Tanzania. Nurs Open.

[34] Fite MB, Roba KT, Oljira L, Tura AK, Yadeta TA (2021). Compliance with Iron and Folic Acid Supplementation (IFAS) and associated factors among pregnant women in Sub-Saharan Africa: a systematic review and meta-analysis. PLoS One.

